# Complementary measurement of nontyphoidal *Salmonella-*specific IgG and IgA antibodies in oral fluid and serum

**DOI:** 10.1016/j.heliyon.2022.e12071

**Published:** 2022-12-15

**Authors:** Sean C. Elias, Esther Muthumbi, Alfred Mwanzu, Perpetual Wanjiku, Agnes Mutiso, Raphael Simon, Calman A. MacLennan

**Affiliations:** a Jenner Institute, Nuffield Department of Medicine, University of Oxford, UK; b KEMRI-Wellcome Trust Research Programme, Kilifi, Kenya; c London School of Hygiene & Tropical Medicine, UK; d University of Maryland, Baltimore, USA

**Keywords:** Nontyphoidal, Oral fluid, IgG, IgA, O-Antigen, Flagellin

## Abstract

**Objectives:**

Immuno-epidemiological studies of orally acquired, enteric pathogens such as nontyphoidal *Salmonella* (NTS) often focus on serological measures of immunity, ignoring potentially relevant oral mucosal responses. In this study we sought to assess the levels and detectability of both oral fluid and serum IgG and IgA to NTS antigens, in endemic and non-endemic populations.

**Methods:**

IgG and IgA antibodies specific for *Salmonella* Typhimurium and *Salmonella* Enteritidis O antigen and phase 1 flagellin were assessed using Enzyme Linked Immunosorbent Assay (ELISA). Paired oral fluid and serum samples were collected from groups of 50 UK adults, Kenyan adults and Kenyan infants. Additionally, oral fluid alone was collected from 304 Kenyan individuals across a range of ages.

**Results:**

Antigen-specific IgG and IgA was detectable in the oral fluid of both adults and infants. Oral fluid antibody increased with age, peaking in adulthood for both IgG and IgA but a separate peak was also observed for IgA in infants. Oral fluid and serum responses correlated for IgG but not IgA. Despite standardised collection the relationship between oral fluid volume and antibody levels varied with age and country of origin.

**Conclusions:**

Measurement of NTS-specific oral fluid antibody can be used to complement measurement of serum antibody. For IgA in particular, oral fluid may offer insights into how protective immunity to NTS changes as individuals transition with age, from maternal to acquired systemic and mucosal immunity. This may prove useful in helping to guide future vaccine design.

## Highlights

• Nontyphoidal *Salmonella*-specific IgG and IgA was detected in the oral fluid of adults and infants.
• Oral fluid antibody increases with age, peaking in adulthood.
• O-antigen- and flagellin-specific oral fluid and serum IgG but not IgA responses correlate.

## 1 Introduction

Nontyphoidal *Salmonella* (NTS), particularly *Salmonella* Typhimurium (Group B *Salmonella*, O:4/O:4,5, H1-i) and *Salmonella* Enteritidis (Group D *Salmonella*, O:9, H1-g/m) are major causes of invasive disease in Sub-Saharan Africa that usually manifests as bacteraemia [1]. This contrasts with the self-limiting, acute gastroenteritis observed with other NTS serovars and the enteric fever associated with *Salmonella* Typhi and Paratyphi [2]. Invasive NTS disease most commonly affects those aged 6 months-3 years, with cases peaking around 1 year of age, but is also observed in adults with HIV infection [1]. Unlike *Salmonella* Typhi, there are currently no licenced vaccines that are effective against nontyphoidal *Salmonella*.

Immuno-epidemiological studies from Africa support the concept that serum antibody acquisition with age may provide protection against invasive NTS disease [3, 4], whilst maternal antibody has been suggested to play a protective role in infants less than 6 months old [3]. It has been proposed that O antigen specific IgG antibody may contribute to protection, as shown in vitro through complement-dependent bactericidal activity [3, 5, 6] and in vivo by passive transfer studies [7].

Despite these observations, there are currently no confirmed correlates of protection against invasive NTS disease in humans. Whilst a number of studies have investigated the role of the mucosal innate immune response in protection against invasive NTS serovars [8, 9], few have looked into the role of the mucosal antibody response. NTS exposure, particularly to invasive serovars is predominantly via the oral route from contaminated water or food [10, 11]. As such, antibodies in oral fluid and other mucosal sites may play an important protective role providing a first barrier of defence against NTS following exposure. Alternatively, an absence of such a mucosal response, particularly in the gut might also contribute to failure to control translocation of invasive NTS serovars which display unique interactions with host immune components [12, 13, 14]. Whilst comparisons can be made to proposed mechanisms of protection against invasive *Salmonella* Typhi disease [15], relevance is limited by the absence of the Vi-polysaccharide on the vast majority of NTS serovars.

Oral fluid is composed of a number of fluids of different origins, each contributing differently to its antibody composition. Gingival crevicular fluid is derived from periodontal tissue located between the lower gum and cheek. It is composed of serum and locally generated materials such as tissue breakdown products, inflammatory mediators, and antibodies originating from the serum. Saliva, in contrast, originates from the parotid, submandibular and sublingual salivary glands [16, 17, 18]. B cell populations independent to those found in blood have been shown to contribute dimeric secretory IgA from these salivary glands, but not IgG which is found only in negligible levels in saliva [18, 19]. One difficulty in working with oral fluid can be dissecting the relative contribution of antibody from these individual locations, despite the observation that secretory IgA (sIgA) has also been shown to be in mostly dimeric form in contrast to monomeric IgA in serum [18].

Specific oral fluid antibodies, in particular IgG and IgA have been identified against numerous bacterial [20, 21, 22, 23] and viral pathogens [24, 25, 26]. IgG in oral fluid has commonly been shown to correlate significantly with serum IgG [21, 23, 24]. For IgA the same relationship has also been observed but varies in significance [20, 22, 23], likely due to its numerous origins. In studies of some oral pathogens such as *E. coli*, antibody responses in the salivary glands were shown to correlate better with gut mucosal immune responses than serum responses [20], however this is not always a clear cut relationship [18]. Oral fluid and salivary antibodies have been identified in newborns [22] and infants and have been shown to increase with age suggesting acquisition over time [17]. In older adults, a decrease in salivary antibodies has been proposed as a possible mechanism for increased susceptibility to bacterial infection [27]. Oral fluid may therefore provide additional data to support new correlates of protection, susceptibility and carriage for NTS in different age groups.

In young children, obtaining serum through venepuncture can be stressful to both child and parent. As a result, patient retention or recruitment of healthy subjects can be difficult, as parental consent is often withdrawn once their child has recovered from an illness, or is not given in the first place. The method of oral fluid collection is non-invasive, making it safer for use in individuals with acute disease such as with Ebola virus [24] or co-infections such as with HIV and has been shown to be well received during the consenting process, particularly with adults consenting on behalf of their child [28]. In most studies to date oral fluid collection has been used to complement rather than replace serum collection for measuring antibody responses against systemic pathogens.

In this study we sought to assess the value of oral fluid for measuring NTS-specific antibody. Using IgG and IgA standardised ELISAs, we compared antibody response in both oral fluid and serum across different populations and age groups, with a focus on individuals from a representative Low- and Middle-Income Country where invasive and diarrhoeal NTS disease are endemic.

## 2 Materials and methods

### 2.1 Study sites

In the UK, paired blood and oral fluid samples were collected from 50 heathy adults at the Centre for Clinical Vaccinology and Tropical Medicine (CCVTM) in Oxford.

In Kenya, sample collection was split into two phases. In phase one, paired blood and oral fluid samples were collected from 50 healthy adults and 50 healthy infants at local health facilities in the coast town of Kilifi, located north of Mombasa. In phase two oral fluid samples alone were collected from 304 individuals across five different age groups (1–12m, 13m-4y, 5-14y, 15-54y & 55y+) at participant's homes. Across both Kenyan cohorts, individuals within the age-groups specified were selected randomly from four local districts (Junju, Kilifi North, Ganze and Kilifi South) using the Kilifi Health and Demographic Surveillance System (KHDSS) [29].

### 2.2 Sample collection

10 mL of blood from adults and 5 mL from infants were collected by qualified health workers into a plain Vacutainer tube and allowed to clot naturally and stored at room temperature prior to processing. In Kenya, samples were stored and transported from the field sites to the laboratory in temperature controlled cool boxes. At both sites, samples were collected in the morning or early afternoon to ensure processing the same working day in the laboratory. Blood samples were centrifuged at 600g for 10 min before serum was carefully removed and divided equally between a minimum of two 2 mL cryovials. Samples were stored at -80°C until required.

**Figure 1:**
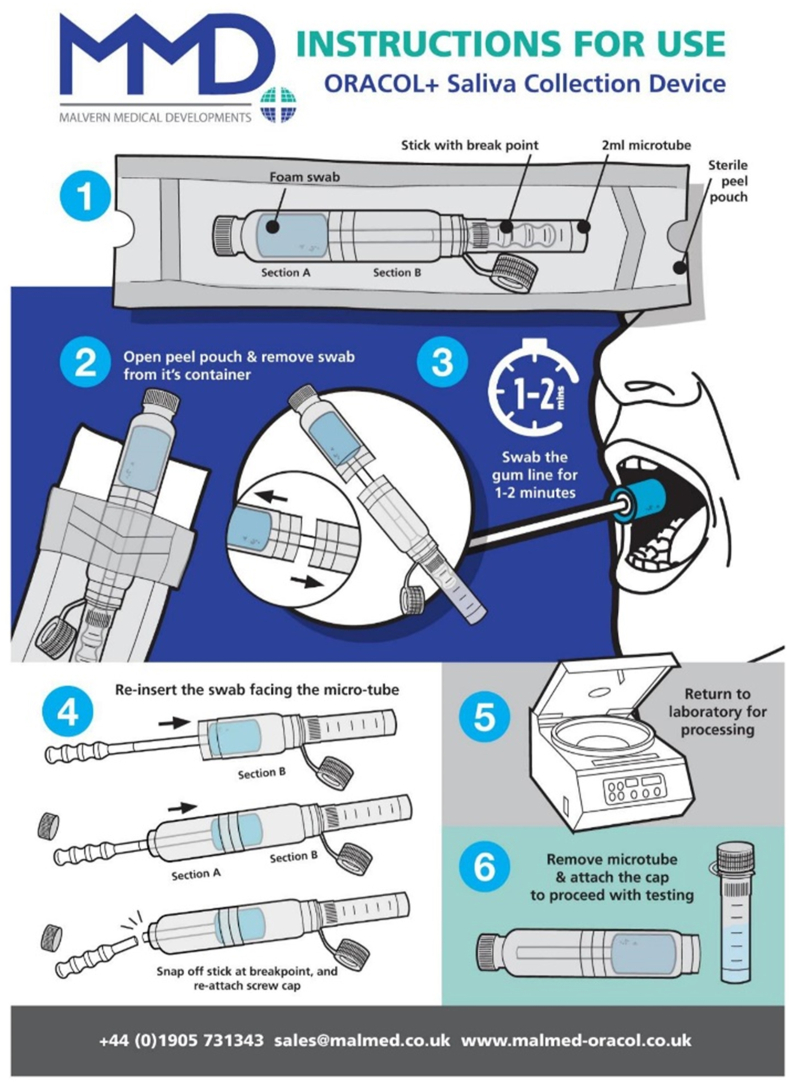
Oracol+ Saliva Collection Device Instructions for Use. Published with permission - Malvern Medical Developments Ltd, UK.

A single oral fluid sample was collected using an Oracol+ oral swab (Figure 1 https://www.malmed-oracol.co.uk/oracol-plus/). In a review of existing literature we found little standardisation in the collection and storage of oral fluid even using the same collection devices [28]. Oracol+ devices were selected over the original base model as the addition of a built in microtube offered improved sample measurement and sterility for field collection. Sample collection involved rubbing the soft sponge swab between the lower gum and cheek for 1–2 min to collect primarily crevicular fluid. Adults either swabbed themselves or were swabbed by a health worker. Infants and children were swabbed by a health worker. Each Oracol+ oral swab was centrifuged at 600g for 5 min to release the fluid into the built-in microfuge tube. Oral fluid samples were centrifuged a second time at 5500g for 5 min to pellet cells and debris. Using a pipette, volume was measured and the cell-free oral fluid was transferred to a fresh cryovial. An equal volume of transport medium [24] (PBS containing 10% FBS, 0.2% Tween 20, 0.5% Geneticin, 0.2% Fungizone) was added to stabilise the sample resulting in a 1 in 2 sample dilution. Samples were stored at -80 °C until required.

### 2.3 Laboratory protocols

Standardised ELISAs were developed using published methodology [30]. ELISAs were performed at site of sample collection in Oxford, UK, or Kilifi, Kenya. A series of measures were put in place to ensure comparability of results generated at the two sites. Firstly, all reagents used in the study were ordered together and shipped from the Oxford site to Kenya. At the Kenyan site, ELISA training was provided by the lead UK and Kenyan scientist who had performed the assay at both sites and were involved in the original assay development. A selection of Kenyan sera were tested at both sites to ensure inter-site variability and user variability was minimal.

O-antigen, O:4,5 (CVD1925) and phase 1 flagellin (CVD19255FliC) from *S.* Typhimurium and O antigen, O:9 (CVD1943) and phase 1 flagellin (CVD1943 FliC) from *S.* Enteritidis, were generated as described [31, 32, 33]. Antigen stocks (between 5-8.5 mg/mL) were diluted to 5 μg/mL in coating buffer (5.3g Na_2_CO_3_ & 4.2g NaHCO_3_ per litre dH_2_0, pH 9.6) and 50μL added per well to an ELISA plate. Plates were incubated overnight at 5 °C then blocked for 1 h using preformulated 1% Casein in PBS (Thermo Scientific). Human test sera were diluted in the same Casein buffer to a starting concentration of 1 in 50 and further diluted in sequential 10-fold dilutions where antibody levels were above the linear portion of the ELISA standard curve. The standard curve and internal controls were created from an in-house human control serum containing antibodies to all four antigens. The positive control was pooled serum from Malawian donors containing antibodies to all four antigens. Plates were washed using PBS containing 0.1% Tween then sera added to triplicate wells and incubated at room temperature for 2 h. Plates were again washed before addition of 50μL per well HRP secondary antibody diluted 1 in 1000 (Polyclonal Goat Anti-Human IgA α-chain specific antibody, Sigma) or 1 in 2000 (Polyclonal Goat Anti-Human IgG γ-chain specific antibody, Sigma) for 1 h then washed 6 times with PBS/0.05%Tween. Plates were developed with 100μl of highest sensitivity TMB solution (AbCam) and stopped with 100μl of Stop Solution (AbCam) once the internal control reached an O.D of 1. Plates were read at 450nm and data analysed using GenV ELISA software. Internal and positive control readings were assessed to ensure they were within 20% of expected values. For plates where control readings fell outside this range, the ELISA was repeated. CVs were determined from individual serum triplicates and repeated where >20%.

Oral fluid ELISAs were performed as for serum ELISAs but using 1% BSA in PBS as dilution buffer. Oral fluid was tested at a final dilution of 1 in 50 for IgA ELISA and 1 in 10 for IgG ELISA.

### 2.4 Statistical analysis

Data were analysed using GraphPad Prism version 9 for Windows (GraphPad Software Inc., California, USA). Groups analyses were performed using Kruskal-Wallace with Dunns multiple comparison tests. Correlations were assessed using Spearman’s Rank test.

### 2.5 Study approval

For the UK, ethics approval was provided by the Medical Sciences Interdivisional Research Ethics Committee (IDREC). For Kenya, ethics approval was first provided by Oxford Tropical Research Ethics Committee (OxTREC) and by the Kenyan Medical Research Institute (KEMRI) Scientific and Ethics Review Unit (SERU). Informed consent was obtained from all participants or their parents or guardians.

## 3 Results

### 3.1 Study population and age distribution

Study participants for specific groups were recruited from either the UK, or Kenya in one of two phases (Table 1). For UK adults, and Kenyan adults in phase one, the mean age of participants was 32 years for both groups. Sex ratios across the two groups were similar (36/64 & 30/70 male to female ratio). Among Kenyan infants in phase one the mean age of participants was 253 days old, with a male to female ratio of 48/52. For Kenyans in phase two the mean ages in each group were; 240 days (1–12m), 2 years 10 months (13m-4y), 10 years 3 months (5-14y), 31 years (15y-54y), 69 years (55y+), with a male to female ratio of 46/54.

**Table 1:** Summary of study population, age & sex distribution and sample collection differences across different study groups.

Study Group	Recruitment Age Range	N =	Mean Age	Median Age	Male/Female	Blood	Oral Fluid
UK Adults	18-55y	50	32y	30y	36/64	Yes	Yes
Kenya Adults (Phase 1)	18-55y	50	32y	31y	30/70	Yes	Yes
Kenya Infants (Phase 1)	1–12m	50	253d	249d	48/52	Yes	Yes
Kenya Mixed Age (Phase 2)	1–12m (Infants) 13m-4y (Younger Children) 5-14y (Older Children) 15-54y (Adults) 55y+ (Elderly)	59 72 56 62 55	240d 2y10m 10y3m 31y 69y	235d 2y9m 10y6m 33y 69y	46/54	No	Yes

### 3.2 Optimisation of oral fluid collection and standardised oral fluid ELISA

Assay standardisation was performed using samples from the UK adult population. Oral fluid collection volumes varied significantly between individuals ranging from 60μL to 1500μL (GMT 431μL , 95%CI 343μL, 542μL) giving final volumes of 120–3000μL after a 1 in 2 dilution of transport medium. In the Kenyan phase 1 population, smaller volumes were obtained, with initial volumes of 6μL to 800μL in adults (GMT 260μL, 95%CI 203μL/333μL) and 40μL to 720μL in infants (GMT 208μL, 95%CI 173μ/250μ), which also doubled with addition of transport medium.

Performing eight oral fluid ELISAs (IgG and IgA for O:4,5, O:9, phase 1 flagellin (FliC) from *S*. Typhimurium and *S*. Enteritidis) at a 1 in 2 dilution requires a minimum volume of 1200μL (150 μL per ELISA). During preliminary laboratory work, it was established that a starting assay dilution of 1 in 10 for IgG ELISAs (15μL per ELISA) and 1 in 50 dilution for IgA ELISAs (3μL per ELISA), ensured that all assays could be conducted with most available volumes, good initial measurement of detectable antibody, and excess volume for repeats. In total there were 5 Kenyan adults and 2 Kenyan children with missing data for one or more antigen due to insufficient volume. The difference between IgG and IgA was in large part due to the ease of detection of IgA in oral fluid compared with IgG.

IgA to all antigens was detected in oral fluid from all UK adults, with the exception of one adult with undetectable IgA to O:4,5. For many UK adults, even using a 1 in 10 dilution, IgG to one or more antigen was not detectable. To gauge the limits of assay sensitivity, samples with undetectable IgG at the starting assay dilution were retested at reduced dilutions of 1 in 2, where sufficient sample was available. Only for IgG, and in particular O:4,5 IgG, did we find a notable increase in detection of antibody when repeating ELISA using a dilution of 1 in 2 (Figure S1). It was not possible to retest ELISA at reduced dilutions with most Kenyan adult and infant oral fluid samples due to insufficient sample volume after initial testing and repeats at initial dilutions.

### 3.3 Profiles of NTS O-antigen- and flagellin-specific IgG and IgA antibody in the oral fluid and serum of UK and Kenyan Adults and Kenyan Infants

**Figure 2:**
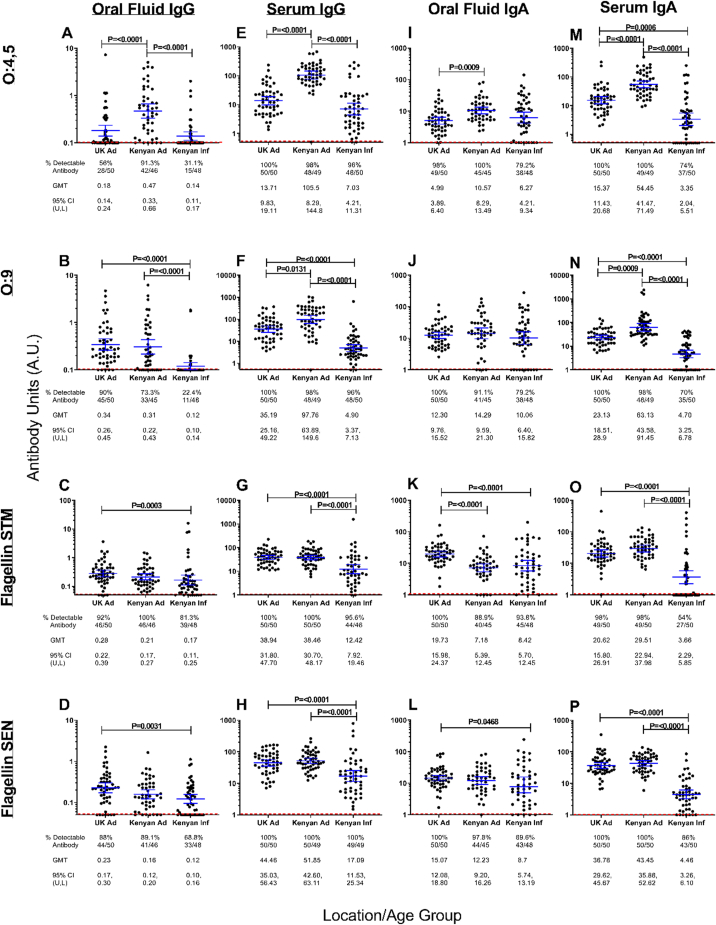
Differences in level and detectability of antigen specific oral fluid and serum IgG and IgA in UK adults, Kenyan adults and Kenyan infants. O:4,5, O:9, S. Typhimurium (STM) flagellin- and S. Enteritidis (SEN) flagellin-specific oral fluid IgG (**A-D**) and IgA (**E-F**) were detected using standardised oral fluid ELISA. O:4,5, O:9, S. Typhimurium (STM) flagellin- and S. Enteritidis (SEN) flagellin-specific serum IgG (**I-L**) and IgA (**M-P**) were detected using standardised serum ELISA. ELISA titres reported as antibody units (A.U). Threshold for limit of detection for each assay indicated by dotted red line. Geomean with 95% CI, and P value shown. Also see Table S1. Groups analysis performed using Kruskal-Wallace with Dunns multiple comparison tests. Results shown are for sample dilutions of 1 in 10 for IgG and 1 in 50 for IgA as standard.

We first investigated the effect of age and location on nontyphoidal *Salmonella* antibody levels using sera from UK adults, and Kenyan adults and children collected in phase 1 (Figure 2, Table S1).

#### 3.3.1 Oral fluid IgG

Differences in age and location had a greater impact on O-antigen antibody levels compared with flagellin antibody levels (Figure 2, column 1). Kenyan adults had significantly higher oral fluid O:4,5 IgG levels and a significantly greater proportion of individuals with detectable oral fluid O:4,5 IgG (0.47 A.U., 91.3%) compared with both UK adults (0.18 A.U., p < 0.0001, 56%) and Kenyan infants (0.14 A.U., p < 0.0001, 31.1%) (Figure 2A). In contrast, there was no significant difference between oral fluid O:9 IgG levels in UK (0.34 A.U.) or Kenyan adults (0.31 A.U.), but surprisingly a lower proportion of Kenyan adults had detectable antibody (73.3%) compared with UK adults (90%) (Figure 2B). For Kenyan infants, oral fluid O:9 IgG levels are significantly lower than both UK and Kenyan adults (p < 0.0001 for both comparisons) and a notably lower proportion have detectable antibodies (22.4%). Oral fluid *S.* Typhimurium and *S*. Enteritidis flagellin IgG levels were similar across all groups (Figure 2 C, D). Kenyan Infant oral fluid flagellin IgG levels were significantly lower compared with those in UK adults for both *S.* Typhimurium (0.17 vs 0.28 A.U., p = 0.0003) and *S.* Enteritidis (0.12 vs 0.23 A.U., p = 0.0031). However, Kenyan adult oral fluid IgG levels to both forms of flagellin (0.21 & 0.16 A.U.) were not significantly different from levels in either UK adults or Kenyan infants. There were less Kenyan infants with detectable oral fluid flagellin IgG compared with both UK and Kenyan adults, for both *S.* Typhimurium flagellin (81.3% vs 92% & 100%) and *S.* Enteritidis flagellin (68.8% vs 88% & 89.1%).

#### 3.3.2 Serum IgG

We also observed differences in serum IgG levels to NTS antigen between groups in an antigen-dependent manner (Figure 2, column 2), similar to the differences seen with oral fluid. Kenyan adults had significantly higher serum O:4,5 IgG levels (105.5 A.U.) compared with both UK adults (13.71 A.U., p < 0.0001) and Kenyan infants (7.03 A.U., p < 0.0001) (Figure 2E). Kenyan adults also had significantly higher O:9 serum IgG levels (97.76 A.U.) compared to both UK adults (35.19 A.U., p = 0.0131) and Kenyan infants (4.90 A.U., p < 0.0001) (Figure 2F). Unlike for serum O:4,5 IgG, UK adults had significantly higher serum O:9 IgG than Kenyan infants (p < 0.0001). In contrast to oral fluid IgG levels, almost all individuals had detectable serum IgG to all antigens. Only 1 Kenyan adult and 2 Kenyan infants had undetectable levels of O:4,5 and O:9 serum IgG. Kenyan infants had significantly lower levels of *S.* Typhimurium (12.42 A.U.) and *S.* Enteritidis flagellin IgG (17.09 A.U.) compared with both UK adults (38.94 A.U. and 44.46 A.U. respectively, p < 0.0001 for both comparisons) and Kenyan Adults (38.94 A.U. and 51.85 A.U. respectively, p < 0.0001 for both comparisons) (Figure 2 G, H). Out of all individual tested, only one Kenyan infant had undetectable antibody for *S.* Enteritidis flagellin.

#### 3.3.3 Oral fluid IgA

There were fewer differences in oral fluid NTS antigen IgA levels between groups, compared with oral fluid IgG levels. This is in part due to relatively higher levels of oral IgA compared with oral IgG in Kenyan infants. We observed significantly lower levels of oral O:4,5 IgA in UK adults (4.99 A.U.) compared with Kenyan adults (10.57, p = 0.0009) but not Kenyan infants (6.27 A.U.) (Figure 2I). Despite no significant difference in oral fluid O:4,5 IgA levels compared with UK adults, Kenyan infants had a notably lower proportion of individuals with detectable antibody (79.2%) compared to UK adults (98%) and Kenyan adults (100%). No significant difference in oral fluid O:9 IgA levels were observed across the three groups (12.3, 14.29 & 10.06 A.U. respectively) but as for O:4,5, Kenyan infants had a notably lower proportion of individuals with detectable oral fluid O:4,5 IgA (79.2%) compared to UK adults (100%) and Kenyan adults (91.1%) (Figure 2J). Interestingly, UK adults had significantly higher levels of oral fluid *S.* Typhimurium flagellin IgA (19.73 A.U.) compared with both Kenyan adults (7.18 A.U., p = <0.0001) and Kenyan infants (8.42 A.U., p < 0.0001) (Figure 2K). Similarly, UK adults had significantly higher levels of *S.* Enteritidis flagellin IgA (15.07 A.U.) compared with Kenyan infants (8.7 A.U., p = 0.0468) but not Kenyan adults (12.23 A.U.) (Figure 2L). There was little difference between the proportions of individuals in each group with detectable oral fluid flagellin antibodies for both serotypes.

#### 3.3.4 Serum IgA

Serum IgA profiles across groups largely resemble those observed for serum IgG. Kenyan adults had significantly higher serum O:4,5 IgA levels (54.45 A.U.) compared with both UK adults (15.37 A.U., p= <0.0001) and Kenyan infants (3.35 A.U., p= <0.0001) (Figure 2M). Levels in UK adults were also significantly higher than in Kenyan infants (p = 0.0006). Kenyan adults had significantly higher serum O:9 IgA levels (63.13 A.U.) compared to both UK adults (23.13 A.U., p = 0.0009) and Kenyan infants (4.70 A.U., p= <0.0001) and levels in UK adults were also significantly higher than Kenyan infants (p= <0.0001) (Figure 2N). With the exception of one Kenyan adult, all adults had detectable serum IgA against both O-antigens. However for Kenyan infants only 74% had detectable serum O:4,5 IgA and 70% serum O:9 IgA. Kenyan infants had significantly lower levels of *S.* Typhimurium flagellin IgA (3.66 A.U.) compared with both UK adults (20.62 A.U., p= <0.0001) and Kenyan Adults (29.51 A.U., p= <0.0001) (Figure 2O). A similar pattern was observed for *S.* Enteritidis serum flagellin IgA (Kenyan infants 4.46 A.U., UK adults 36.78 A.U., Kenyan adults 43.45 A.U., p=<0.0001 for both comparisons) (Figure 2P). Almost all adults had detectable serum IgA against both flagellins, but only 54% of Kenyan infants had detectable *S*. Typhimurium serum flagellin IgA and 86% detectable *S*. Enteritidis serum flagellin IgA.

#### 3.4 Oral fluid and serum responses correlated for IgG but not for IgA

**Figure 3:**
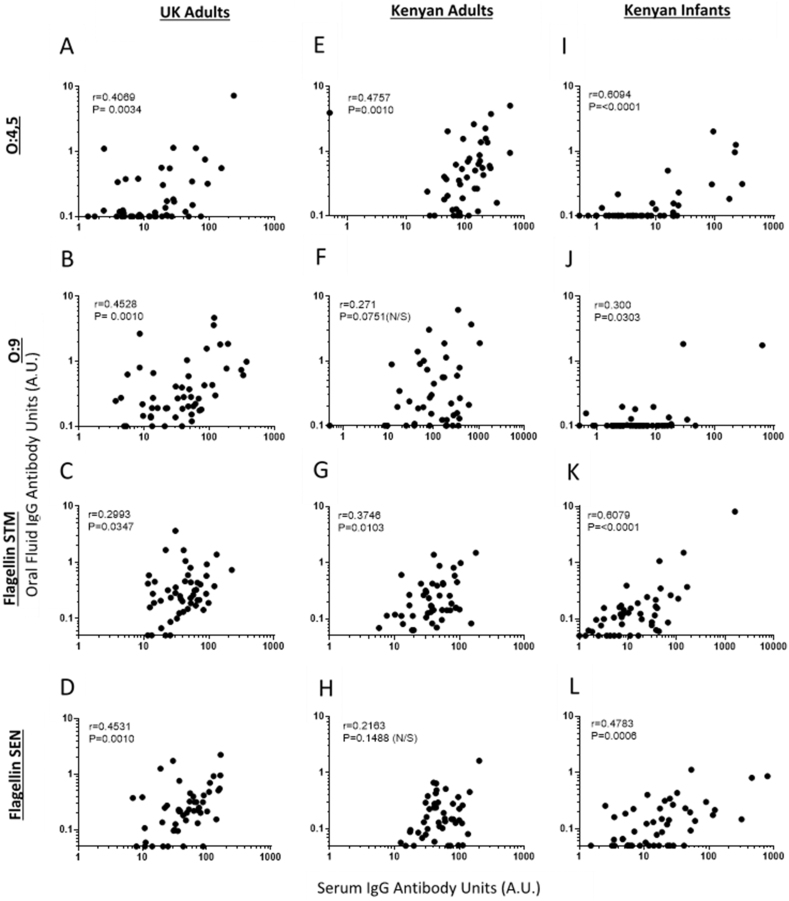
Correlations between antigen specific IgG levels in oral fluid and serum UK adults (**A-D**), Kenyan Adults (**E-H**) and Kenyan Infants (**I-L**). Antigens: O:4,5 (row 1), O:9 (row 2), flagellin of *Salmonella* Typhimurium (STM) (row 3), flagellin of *Salmonella* Enteritidis (SEN) (row 4). Spearman’s Rank test with r and P values shown. Antibody Units (A.U.).

Next, we investigated whether oral fluid IgG and IgA correlated with serum IgG and IgA respectively. There were positive correlations between oral fluid IgG and serum IgG for all four antigens for UK adults and Kenyan infants, and for *S*. Typhimurium antigens; O:4,5 (Figure 3 A, E, I) and flagellin (Figure 3 C, G, K), but not *S*. Enteritidis antigens; O:9 (Figure 3 B, F, J) and flagellin (Figure 3 D, H, L) for Kenyan Adults. Of note, low but detectable levels of serum IgG to specific antigens could be measured for the many Kenyan infants lacking detectable oral fluid IgG for those antigens.

**Figure 4:**
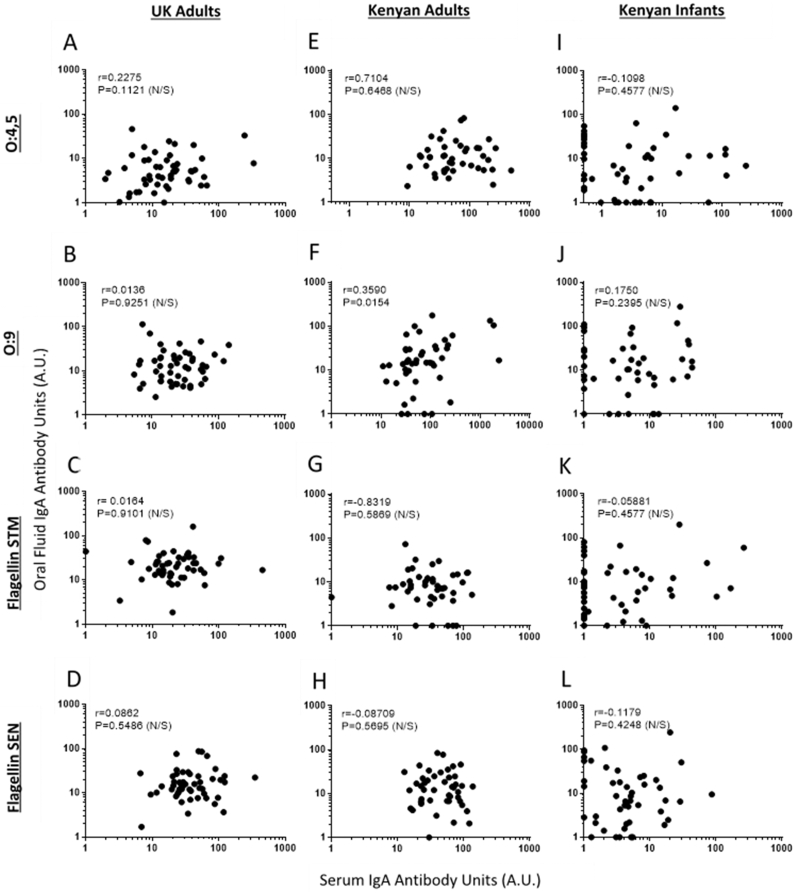
Correlations between antigen-specific IgA in oral fluid and serum UK adults (**A-D**), Kenyan Adults (**E-H**) and Kenyan Infants (**I-L**). Antigens: O:4,5 (row 1), O:9 (row 2), flagellin of *Salmonella* Typhimurium (STM) (row 3), flagellin of *Salmonella* Enteritidis (SEN) (row 4). Spearman’s Rank test with r and P values shown. Antibody Units (A.U.).

With the exception of O:9 antigen for Kenyan adults, no correlations were observed between oral fluid IgA and serum IgA for any antigens, in any of the three population groups (Figure 4 A-L). As previously mentioned, almost all adults from the UK and Kenya had detectable specific IgA to all NTS antigen in both oral fluid and serum. For the many Kenyan infants lacking detectable serum IgA to an NTS antigen, oral fluid IgA to that antigen was present and measurable. Interestingly, for those infants lacking detectable antigen-specific oral fluid IgA, serum IgA was often detectable.

### 3.5 Relationship between oral fluid volume and Salmonella antibody levels varied with age and location

**Figure 5:**
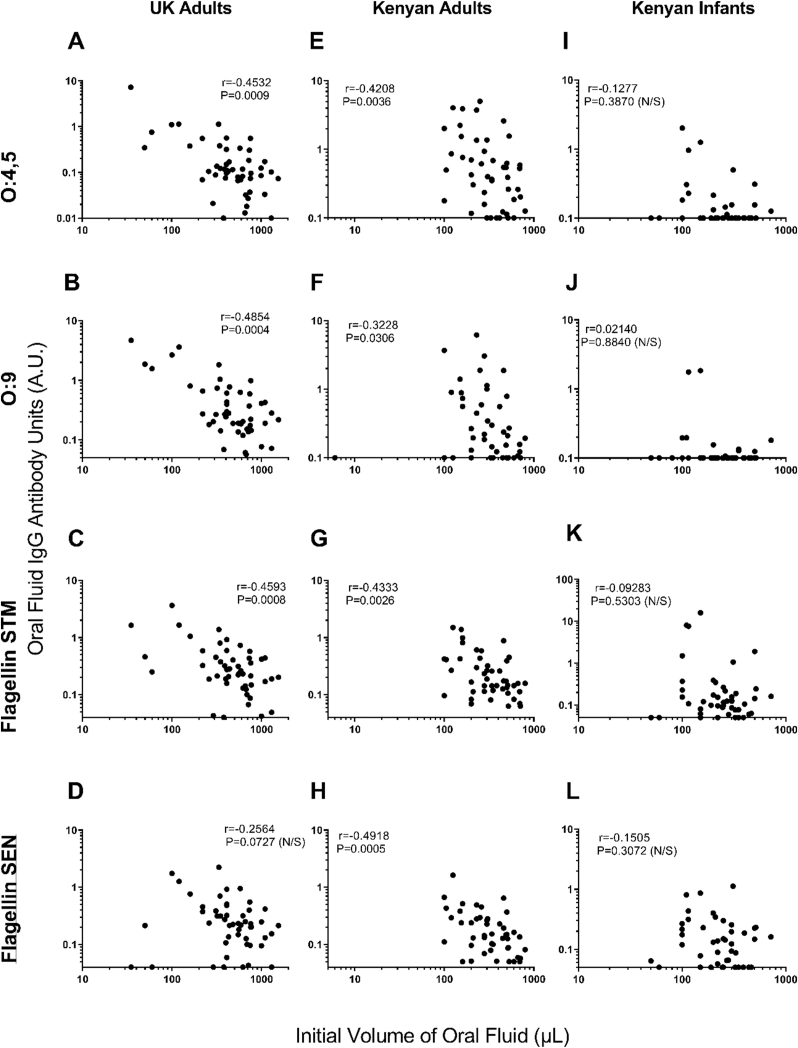
Correlations between antigen-specific IgG levels and initial volume of oral fluid collected. UK adults (**A-D**), Kenyan Adults (**E-H**) and Kenyan Infants (**I-L**). Antigens: O:4,5 (row 1), O:9 (row 2), flagellin of *Salmonella* Typhimurium (STM) (row 3), flagellin of *Salmonella* Enteritidis (SEN) (row 4). Spearman’s Rank test with r and P values shown. Antibody Units (A.U.).

**Figure 6:**
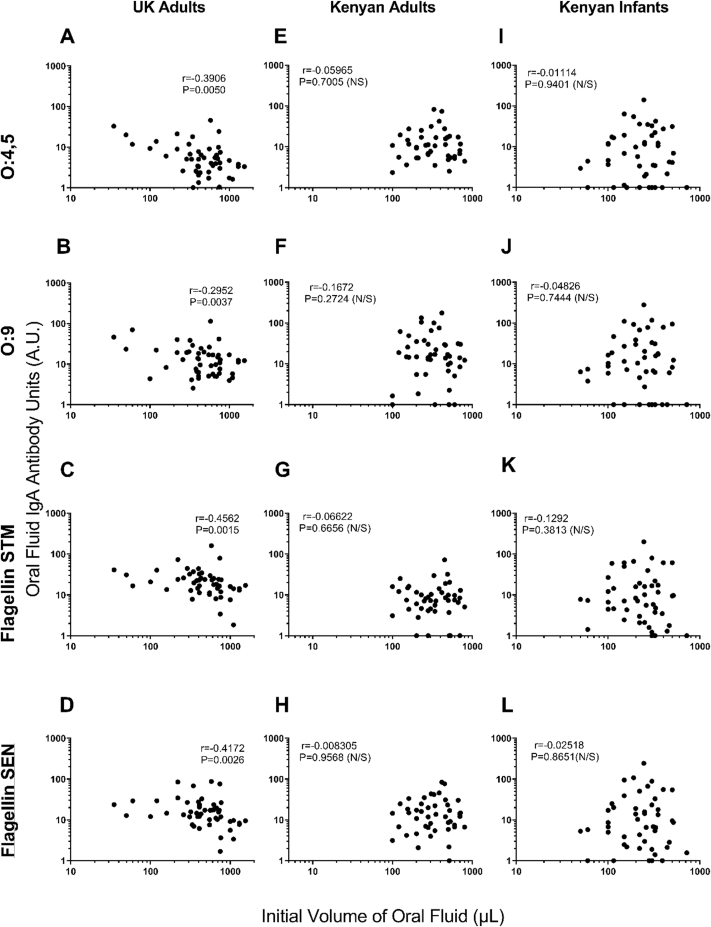
Correlations between antigen specific IgA levels and initial volumes of oral fluid collected. UK adults (**A-D**), Kenyan Adults (**E-H**) and Kenyan Infants (**I-L**). Antigens: O:4,5 (row 1), O:9 (row 2), flagellin of *Salmonella* Typhimurium (STM) (row 3), flagellin of *Salmonella* Enteritidis (SEN) (row 4). Spearman’s Rank test with r and P values shown. Antibody Units (A.U.).

As mentioned, oral fluid volumes collected from individuals varied. In UK adults GMT antibody inversely correlated with oral fluid volume for all antigens for IgG, with the exception of *S*. Enteritidis flagellin (Figure 5 A-D), and all antigens for IgA (Figure 6 A-D). In Kenyan adults this inverse correlation was also observed across for all antigens for IgG (Figure 5 E-H), but not IgA (Figure 6 E-H). In Kenyan infants there was no correlation between oral fluid antibody levels and volume for either IgG or IgA (Figures 5 and 6 I-L).

### 3.6 Oral fluid nontyphoidal Salmonella IgG and IgA antibody profiles with age in Kenya

**Figure 7:**
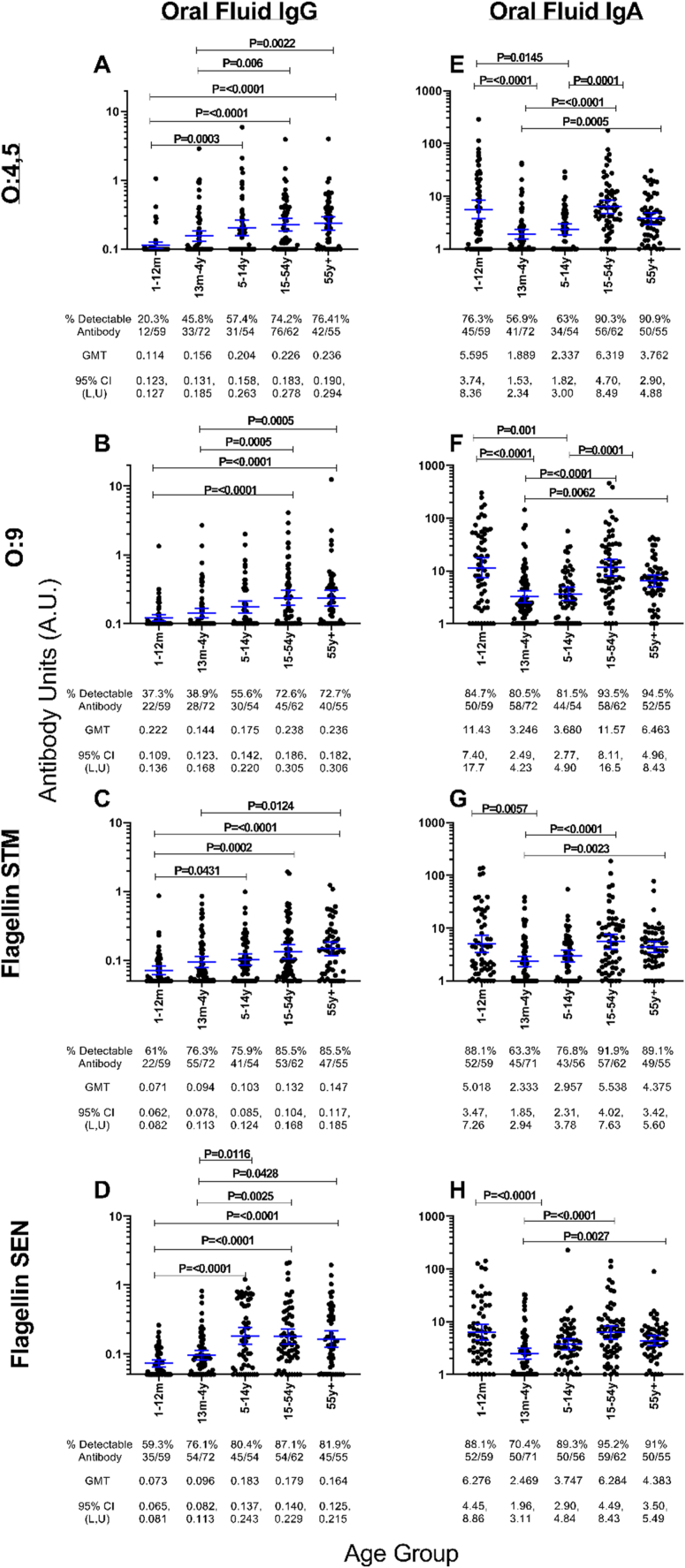
**Differences in levels and detectability of antigen-specific oral fluid IgG and IgA with age.** Study population was subdivided by age group (Infants: 1–12months, Young Children: 13months-4 years 11 months, Older Children: 5 years -14y 11months, Adults: 15–54 years 11 months, Elderly: 55 years and above). O:4,5 (**A-B**), O:9 (**C-D**), S. Typhimurium flagellin (**E-F**), S. Enteritidis flagellin (**G-H**). ELISA titres reported as antibody units (A.U). % of individuals with detectable antibody, Geomean with 95% CI shown and P values shown. Also see Table S2. Groups analysed using Kruskal-Wallis with Dunns multiple comparison tests.

Levels of oral fluid antibodies to NTS antigens increased with age among Kenyans, across all antigens tested, with differences observed for IgG and IgA (Figure 7, Table S2).

#### 3.6.1 Oral fluid IgG

Oral fluid IgG and the percentage of individuals with detectable IgG to all NTS antigens gradually increases with age and peaks in adulthood (Figure 7 A-D). IgG levels in older children aged 5–14 years were significantly higher compared with infants aged 1–12 months for O:4,5 (p = 0.0003) and both *S*. Typhimurium flagellin (p = 0.0431) and *S*. Enteritidis flagellin (p= <0.0001). Oral fluid O:9 IgG was significantly higher in adults compared with infants (p < 0.0001). No significant differences were observed between older children, adult and elderly groups across all antigens. Fewer infants had detectable oral fluid IgG for NTS O-antigens (O:4,5 20.3%; O:9 37.3%) compared with NTS flagellin (*S*. Typhimurium 61%; *S*. Enteritidis 59.3%). Kenyan adults had similar proportions of detectable oral fluid IgG for O:4,5 (74.2%) and O:9 (72.6%). The proportion of individuals with detectable oral fluid IgG against flagellin antigens were notably higher than for O antigens but similarly comparable to one another (*S*. Typhimurium 85.5%, *S*. Enteritidis 87.1%).

#### 3.6.2 Oral fluid IgA

Oral fluid IgA and the percentage of individuals with detectable IgA to all NTS antigens gradually increases with age and peaks in adulthood, but with the major exception that a large percentage of infants (O:4,5 76.3%, O:9 84.7%, *S*. Typhimurium 88.1%; *S*. Enteritidis 88.1%) had detectable and on average higher IgA levels (Figure 7 E-H). O:4,5 and O:9 IgA levels in infants were significantly higher than both young children aged 13m-4 years (p < 0.0001 for both antigens) and older children aged 5–14years (O:4,5 p = 0.0145, O:9 p = 0.001), but were not significantly different to adults. Infant *S*. Typhimurium and *S*. Enteritidis flagellin IgA levels were significantly higher than those observed in young children (p = 0.0057 & <0.0001 respectively) but not significantly higher than those in older children or adults. O:4,5 and O:9 IgA levels in adults were significantly higher than both younger children (p = 0.0001 for both comparisons) and older children (p < 0.0001 for both comparisons), whilst *S*. Typhimurium and *S*. Enteritidis flagellin IgA levels in adults were only significantly higher compared to younger children (p=<0.0001 for both comparisons). The proportion of Kenyan adults with detectable IgA at the peak of the response were comparably high for all antigens (O:4,5 90.3%, O:9 93.3%, *S*. Typhimurium 91.9% and *S*. Enteritidis 95.2%) and higher than those observed for IgG. A drop in IgA levels, but not the percentage of individuals with detectable IgA were observed for all antigens in the elderly group.

## 4 Discussion

To date the potential role of serum antibody in protection against non-typhoidal *Salmonella* (NTS), and its acquisition with age has been well established [34]. In this study, we aimed to assess the potential value of using oral fluid alongside serum in immunological studies for NTS. To do this, we first established that NTS specific IgG and IgA are detectable in oral fluid, and then assessed potential relationships between oral fluid and serum NTS-specific IgG and IgA.

Direct comparisons between NTS antibody levels in endemic and non-endemic populations are currently under-represented in the literature, but robust functional antibody responses have been previously observed in both populations [36, 37]. In our study there were few significant differences between oral fluid IgG and IgA levels to NTS antigens in the two adult populations. Kenyan adults had significantly higher O:4,5 oral fluid IgG and IgA but no differences were observed for O:9. In Kenya it has been reported that there is a higher prevalence of the O:4,5 *S*. *Typhimurium*serovar compared O:9 *S*. Enteritidis [38, 39] which may help explain this difference. Previously reported differences in O-antigen and whole lipopolysaccharide serovar-specific serum IgG across endemic populations [4, 40, 41] may also reflect differences in local serovar prevalence, though more evidence is required to prove this definitively.

Our second aim was to establish whether there was a clear relationship between oral fluid and serum NTS-specific IgG and IgA. Oral fluid and serum IgG correlated for different NTS antigen and in different subject groups across the study. This suggests IgG in the two media originates from a common source. In contrast, oral fluid and serum NTS-specific IgA did not correlate. Many infants had high levels of oral fluid IgA but absent serum IgA for a specific antigen, or high levels of serum IgA and absent oral fluid IgA. This suggests that IgA in the two media may have different age-associated origins.

At least one previous study has described protective immunity associated with maternal placental NTS specific IgG [3]. Declining maternal IgG at around four months was shown to associate with an increased susceptibility to NTS disease, but this exposure subsequently drove detectable seroconversion in the cohort. The vast majority of oral fluid IgG comes from crevicular fluid which derives directly from circulating blood by passive leakage mainly via gingival crevicular epithelium [19]. In the absence of any other salivary source, a correlation is expected between IgG in oral fluid and serum. However, this does not explain why detectable oral fluid IgG to specific NTS antigens was undetectable in some adults with high levels of corresponding serum antibody. Total IgG is much lower in oral fluid than in serum [19]. An inverse correlation between oral fluid volume and NTS-specific IgG levels was observed for adults but not infants in this study. This has also been reported for other pathogen-specific IgG [42]. High volumes of saliva, which contain virtually no IgG, were found to dilute out crevicular fluid IgG. The combination of lower overall detection of IgG in infant oral fluid, particularly for O antigens, along with a smaller range of oral fluid volumes collected compared with adults, likely limited the ability to detect any correlation between oral fluid sample size and antibody level. Dilution of oral fluid during sample preparation for storage or ELISA is likely to increase the difficulty of detecting low levels of IgG. We confirmed through testing that less dilute oral fluid allowed for greater sensitivity in the ELISA.

Infants and young children in endemic countries are the main targets for future NTS vaccines. The lack of sensitivity for detecting oral fluid IgG in these groups, particularly for the leading vaccine target, O-antigen, makes oral fluid NTS-specific IgG less viable as a replacement for serum NTS-specific IgG, despite good correlation between the two. If future vaccination drives high levels of oral fluid IgG or results in a change in this relationship, it could be worth revisiting oral IgG as a measurement of immunity to NTS.

For other pathogens, correlations between antigen-specific IgA in oral fluid and serum have been described [21, 23] and in such cases oral fluid has been proposed as a potential surrogate for serum. IgA comprises a higher proportion of total antibody in oral fluid compared to IgG [16, 19] and accordingly we observe far fewer individuals with undetectable levels of IgA in oral fluid. It is unlikely therefore that the lack of correlation between oral fluid and serum IgA observed in our study is due to insufficient power to detect a correlation. However oral fluid IgA does not just derive from crevicular fluid. Up to 35% of total oral fluid IgA is proposed to originate from parotid, sublingual, and submandibular saliva [17, 18]. This secretory IgA is mainly dimeric, produced by local B cells and may reflect other populations in either the gut or tonsils [19, 20]. These B cells may be T-independent and primed by local dendritic cell populations based on studies in mice [43]. We observed that individual UK adults with a high volume of oral fluid had lower detectable NTS-specific IgA, as observed with IgG. This suggests that the saliva of UK adults contains little NTS-specific IgA and that such fluid only serves to dilute detectable IgA originating in crevicular fluid [20]. Increased oral fluid volumes observed in Kenyan adults do not inversely correlate with IgA, supporting the idea that their saliva may contain higher levels of NTS-specific IgA compared with UK adults. From our data we cannot however determine the exact origins of NTS IgA. There is also a possibility that some of the IgA detected may be polyreactive secretory IgA rather than NTS-specific [44]. Though secretory IgA has been observed in infant oral fluid, the most likely explanation for raised IgA levels is that IgA is originating from breast feeding [22, 45, 46]. IgA unlike IgG is not specifically carried cross the placenta via the neonatal Fc receptor and so is not expected to be present from birth in serum or consequently crevicular fluid [47]. Infants with high levels of oral IgA typically had no detectable serum IgA, fitting this hypothesis. Additionally, we see a drop in IgA between infants and young children, likely resultant from weaning and reduced breast-feeding frequency.

Our results suggest that NTS-specific oral fluid IgA is best viewed as an independent measure of immunity to that in serum. It is best used to supplement the measurement of systemic IgA, but may yet prove useful as a surrogate for mucosal IgA that is more difficult to measure [20]. As an orally acquired pathogen [48], the buccal cavity it the first port of entry for *Salmonella.* Oral IgA derived from both serum and secretory IgA [19], may play an important role in protection or control of oropharyngeal carriage as with other enteric infections [49, 50, 51, 52], and may correlate directly with gut immunity [18, 20]. This is important, as gut mucosal immunity is difficult to measure beyond the mouth. In contrast to the complement-dependent bactericidal activity provided by serum IgG, mucosal secretory IgA is proposed to function through formation of immunological aggregates through agglutination and plays an important role in preventing gut translocation and localised inflammation which can lead to invasive NTS disease [44, 53, 54]. Another potential measure of gut mucosal immunity is faecal IgA. Faecal IgA may also have implications for faecal carriage and transmission which is not well understood [55, 56]. It may be possible to test IgA in maternal breast milk, infant oral fluid and infant stool and explore sequential antibody transfer and maintenance through the gut [57]. Breast milk derived IgA has been shown to survive through the infant digestive tract [57, 58] and may play a role in limiting NTS carriage in infant stool, alongside a role in probiotic control [59]. Despite these possibilities, investigation of IgA may be less useful in NTS vaccine studies compared to studies of NTS natural infection. Most current candidate NTS vaccines are parenterally administered and induce a primarily systemic immune response but are less effective at inducing mucosal immunity [2]. A protective role for IgA may however support further investigation into orally administered NTS vaccines [60] or maternal vaccination [61].

There are several drawbacks to the approaches used in this study. Addressing these could provide insights and clarifications for some of the ideas presented above. Firstly, there was variability in the sensitivity of some oral fluid NTS ELISA compared to equivalent serum ELISA. For serum ELISA, use of high titre endemic pooled serum or serum from vaccinated individuals as standards are commonly used to improve sensitivity [62]. Oral fluid collected in this study is unlikely sufficient for developing new standards for future studies so high titre alternatives need to be identified. In this study we did show that lower dilutions of oral fluid could be used to improve sensitivity but this came at the expense of excess volumes for repeat or multiple assays. Whilst this is very limiting when looking at multiple antigens associated with natural exposure it may be less of an issue when studying immunity to a single antigen in a vaccine study.

Alternatively, we could address this issue during the sample collection step. Collection of oral fluid as its constituent salivary and crevicular components would firstly help negate the effect of salivary dilution of crevicular antibody and secondly help assess their relative contributions to total NTS-specific antibody [42]. Whilst methods such as collection of passive drool may allow for better collection of saliva, it requires additional consideration such as natural variations in secretory flow rates [19]. To more accurately address relative contributions to oral fluid we may have to look at separation on a molecular rather than physical level. The measurement of total IgG and IgA alongside NTS-specific IgG and IgA in both serum and oral fluid, and presenting these values as a proportion of total antibody may add value [42]. This approach would help allow for differences in volume for oral fluid and permit additional considerations. These include primary IgA immunodeficiencies [63], and IgG hypergammaglobulinemia caused by factors such as HIV [37], which may be prevalent in tested populations. Additionally, we could attempt to measure independently, monomeric IgA (in serum) and dimeric secretory IgA (in saliva or breast milk) to better separate antibody according to origin. Secretory IgA has been shown to be subject to short term variations within individuals, driven potentially by both circadian rhythms [64] and stress [65]. Longitudinal data may be required to understand the complete picture [18].

In this study antibody cross reactivity was also not accounted for. Cross-priming of common flagellin epitopes from different serovars expressing phase 1 and 2 antigens has been well described [66]. In this study we found that Typhimurium- and Enteritidis- specific Flagellin antibody strongly correlated with each other (data not presented, all P values > 0.0001), both for IgG and IgA and in both oral fluid and serum. Whilst assays such as ELISA may detect physical cross reactivity this may not represent functional cross reactivity [67, 68] supporting the use of functional assays in future studies. Use of the hypervariable D2 and D3 domains of Flagellin instead of the whole protein may help address serovar specific responses in future studies. Cross reactivity may also apply to O-antigen [69] and is not restricted to serum or oral fluid alone.

Our results also highlighted limitations in our study design. Firstly we did not directly control for breast feeding before sampling infants [45]. Recent feeding would be expected to result in higher levels of IgA, whilst bottle feeding would be expected to result in lower or undetectable levels of IgA. The decision to breast over bottle feed is often directly linked to a number of socio-economic factors [70]. For some, but not all enteric diseases, breast feeding has been shown to provide a level of protection against infection [71, 72, 73]. Additionally, we did not look at a range of ages in our non-endemic UK population [36]. Inclusion of these two parameters alongside a subdivision of infants by month post birth would allow for more accurate subdivision of breast milk IgA verses mucosal IgA primed by exposure, as well as their relative contributions to protection against NTS disease. We also did not collect samples from the mothers of infants enrolled in the study. Measuring NTS-specific serum IgG would have allowed us to correlate maternal IgG in mothers and infants and assess changes in this relationship over the first year of life [40]. As discussed above, collection of breast milk IgA would have allowed us to establish relationships between antigen-specific IgA in milk and infant oral fluid, but would have required development of breast milk ELISAs in parallel. Finally we did not distinguish between *Salmonella-* positive and negative populations or those with recent infection. There is a possibility that individuals included in this study were NTS carriers. Whilst there is a lack of data supporting a relationship between carriage and serum antibody, we cannot at this stage discount a potential impact on oral fluid antibody.

In conclusion, we suggest a potential future role for oral fluid in the study of immunity to NTS. This should not be in isolation, but as part of a concerted effort to better study localised immunity associated with NTS sites of entry, colonisation, translocation and carriage. This study in particular highlighted that mucosal IgA and in particular secretory IgA may potentially offer valuable additional insights that complement previous work focusing on the role of systemic IgG. There are clear next steps. Collection methods need to be improved to delineate individual components, sensitivity of existing assays need to be improved and new methods developed to investigate functionality. Once established, measuring oral fluid specifically, and mucosal immunity more generally, alongside systemic immunity may help provide opportunities to measure novel correlates of protection associated with both natural- and vaccine-induced immunity that to date we have struggled to find.

## Declarations

### Author contribution statement

Sean C Elias, DPhil: Conceived and designed the experiments; Performed the experiments; Analyzed and interpreted the data; Wrote the paper.

Esther Muthumbi; Alfred Mwanzu; Perpetual Wanjiku; Agnes Mutiso: Performed the experiments.

Raphael Simon: Contributed reagents, materials, analysis tools or data.

Calman A. MacLennan: Analyzed and interpreted the data; Contributed reagents, materials, analysis tools or data; Wrote the paper.

### Funding statement

This work was supported by the Bacterial Vaccines (BactiVac) Network funded by theGCRF Networks in Vaccines Research and Development which was co-funded by the MRC and BBSRC. BactiVac Grant Ref: BVNCP-12.

### Data availability statement

Data included in article/supplementary material/referenced in article.

### Declaration of interests statement

The authors declare no conflict of interest.

### Additional information

No additional information is available for this paper.

## Appendix A Supplementary data

HLY_12071 - Supplementary file.docx HLY_12071 - Supplementary file.docx

Figure S1_V2.tif Figure S1_V2.tif

## References

[1] Gilchrist J.J., MacLennan C.A. (2019). Invasive nontyphoidal Salmonella disease in Africa. EcoSal Plus.

[2] MacLennan C.A., Martin L.B., Micoli F. (2014). Vaccines against invasive Salmonella disease: current status and future directions. Hum. Vaccines Immunother..

[3] MacLennan C.A. (2008). The neglected role of antibody in protection against bacteremia caused by nontyphoidal strains of Salmonella in African children. J. Clin. Invest..

[4] Nyirenda T.S. (2014). Sequential acquisition of T cells and antibodies to nontyphoidal Salmonella in Malawian children. J. Infect. Dis..

[5] Goh Y.S., MacLennan C.A. (2013). Invasive African nontyphoidal Salmonella requires high levels of complement for cell-free antibody-dependent killing. J. Immunol. Methods.

[6] MacLennan C.A. (2014). Antibodies and protection against invasive salmonella disease. Front. Immunol..

[7] Goh Y.S. (2015). Monoclonal antibodies of a diverse isotype induced by an O-antigen glycoconjugate vaccine mediate in vitro and in vivo killing of african invasive nontyphoidal Salmonella. Infect. Immun..

[8] Hurley D. (2014). Salmonella-host interactions - modulation of the host innate immune system. Front. Immunol..

[9] Keestra-Gounder A.M., Tsolis R.M., Baumler A.J. (2015). Now you see me, now you don't: the interaction of Salmonella with innate immune receptors. Nat. Rev. Microbiol..

[10] Anderson C.J., Kendall M.M. (2017). Salmonella enterica serovar typhimurium strategies for host adaptation. Front. Microbiol..

[11] Mbae C. (2020). Factors associated with occurrence of salmonellosis among children living in Mukuru slum, an urban informal settlement in Kenya. BMC Infect. Dis..

[12] Carden S. (2015). Non-typhoidal Salmonella Typhimurium ST313 isolates that cause bacteremia in humans stimulate less inflammasome activation than ST19 isolates associated with gastroenteritis. Pathog Dis.

[13] Okoro C.K. (2015). Signatures of adaptation in human invasive Salmonella Typhimurium ST313 populations from sub-Saharan Africa. PLoS Neglected Trop. Dis..

[14] Ramachandran G. (2015). Invasive Salmonella Typhimurium ST313 with naturally attenuated flagellin elicits reduced inflammation and replicates within macrophages. PLoS Neglected Trop. Dis..

[15] Jin C. (2021). Vi-specific serological correlates of protection for typhoid fever. J. Exp. Med..

[16] Mortimer P.P., P J.V. (1991). Non-invasive virological diagnosis: are saliva and urine specimens adequate substitutes for blood?. Rev. Med. Virol..

[17] Sonesson M. (2011). Salivary IgA in minor-gland saliva of children, adolescents, and young adults. Eur. J. Oral Sci..

[18] Brandtzaeg P. (2007). Do salivary antibodies reliably reflect both mucosal and systemic immunity?. Ann. N. Y. Acad. Sci..

[19] Brandtzaeg P. (2013). Secretory immunity with special reference to the oral cavity. J. Oral Microbiol..

[20] Aase A. (2016). Salivary IgA from the sublingual compartment as a novel noninvasive proxy for intestinal immune induction. Mucosal Immunol..

[21] Heaney J.L.J. (2018). The utility of saliva for the assessment of anti-pneumococcal antibodies: investigation of saliva as a marker of antibody status in serum. Biomarkers.

[22] Nogueira R.D. (2012). Salivary IgA antibody responses to Streptococcus mitis and Streptococcus mutans in preterm and fullterm newborn children. Arch. Oral Biol..

[23] Barnes G.K. (2016). Salivary and serum antibody response against Neisseria meningitidis after vaccination with conjugate polysaccharide vaccines in Ethiopian volunteers. Scand. J. Immunol..

[24] Lambe T. (2016). Detection of vaccine-induced antibodies to Ebola virus in oral fluid. Open Forum Infect. Dis..

[25] Rowhani-Rahbar A. (2009). Antibody responses in oral fluid after administration of prophylactic human papillomavirus vaccines. J. Infect. Dis..

[26] Gammie A., Morris R., Wyn-Jones A.P. (2002). Antibodies in crevicular fluid: an epidemiological tool for investigation of waterborne disease. Epidemiol. Infect..

[27] Heaney J.L. (2015). Salivary functional antibody secretion is reduced in older adults: a potential mechanism of increased susceptibility to bacterial infection in the elderly. J. Gerontol. A Biol. Sci. Med. Sci..

[28] Vyse A.J., Cohen B.J., Ramsay M.E. (2001). A comparison of oral fluid collection devices for use in the surveillance of virus diseases in children. Publ. Health.

[29] Scott J.A. (2012). Profile: the Kilifi health and demographic surveillance system (KHDSS). Int. J. Epidemiol..

[30] Miura K. (2008). Development and characterization of a standardized ELISA including a reference serum on each plate to detect antibodies induced by experimental malaria vaccines. Vaccine.

[31] Baliban S.M. (2017). Development of a glycoconjugate vaccine to prevent invasive Salmonella Typhimurium infections in sub-Saharan Africa. PLoS Neglected Trop. Dis..

[32] Baliban S.M. (2018). Immunogenicity and induction of functional antibodies in rabbits immunized with a trivalent typhoid-invasive nontyphoidal Salmonella glycoconjugate formulation. Molecules.

[33] Simon R. (2014). A scalable method for biochemical purification of Salmonella flagellin. Protein Expr. Purif..

[34] MacLennan C.A., Tennant S.M. (2013). Comparing the roles of antibodies to nontyphoidal Salmonella enterica in high- and low-income countries and implications for vaccine development. Clin. Vaccine Immunol..

[36] Trebicka E. (2013). Role of antilipopolysaccharide antibodies in serum bactericidal activity against Salmonella enterica serovar Typhimurium in healthy adults and children in the United States. Clin. Vaccine Immunol..

[37] Goh Y.S. (2016). Bactericidal immunity to Salmonella in africans and mechanisms causing its failure in HIV infection. PLoS Neglected Trop. Dis..

[38] Muthumbi E. (2015). Invasive salmonellosis in Kilifi, Kenya. Clin. Infect. Dis..

[39] Onsare R.S. (2015). Relationship between antibody susceptibility and lipopolysaccharide O-antigen characteristics of invasive and gastrointestinal nontyphoidal Salmonellae isolates from Kenya. PLoS Neglected Trop. Dis..

[40] de Alwis R. (2019). The role of maternally acquired antibody in providing protective immunity against nontyphoidal Salmonella in urban Vietnamese infants: a birth cohort study. J. Infect. Dis..

[41] Stockdale L. (2019). Cross-sectional study of IgG antibody levels to invasive nontyphoidal Salmonella LPS O-antigen with age in Uganda. Gates Open Res..

[42] Garrison-Desany H. (2021). Adjustments for oral fluid quality and collection methods improve prediction of circulating tetanus antitoxin: approaches for correcting antibody concentrations detected in a non-invasive specimen. Vaccine.

[43] Kataoka K. (2011). Oral-nasopharyngeal dendritic cells mediate T cell-independent IgA class switching on B-1 B cells. PLoS One.

[44] Bioley G. (2017). Plasma-derived polyreactive secretory-like IgA and IgM opsonizing Salmonella enterica typhimurium reduces invasion and gut tissue inflammation through agglutination. Front. Immunol..

[45] Fitzsimmons S.P. (1994). Immunoglobulin A subclasses in infants' saliva and in saliva and milk from their mothers. J. Pediatr..

[46] Kanariou M. (1995). Age patterns of immunoglobulins G, A & M in healthy children and the influence of breast feeding and vaccination status. Pediatr. Allergy Immunol..

[47] Simister N.E., Mostov K.E. (1989). An Fc receptor structurally related to MHC class I antigens. Nature.

[48] Smith S.I., Seriki A., Ajayi A. (2016). Typhoidal and non-typhoidal Salmonella infections in Africa. Eur. J. Clin. Microbiol. Infect. Dis..

[49] Ostergaard P.A. (1976). IgA levels and carrier rate of Haemophilus influenzae and beta-haemolytic streptococci in children undergoing tonsillectomy. Acta Pathol. Microbiol. Scand. C.

[50] Evans C.M. (2011). Nasopharyngeal colonization by Neisseria lactamica and induction of protective immunity against Neisseria meningitidis. Clin. Infect. Dis..

[51] Holmgren J. (1992). Mucosal immunity: implications for vaccine development. Immunobiology.

[52] Goddard F.G.B. (2020). Child salivary SIgA and its relationship to enteric infections and EED biomarkers in Maputo, Mozambique. Int. J. Environ. Res. Publ. Health.

[53] Moor K. (2017). High-avidity IgA protects the intestine by enchaining growing bacteria. Nature.

[54] Richards A.F. (2021). Recombinant human secretory IgA induces Salmonella typhimurium agglutination and limits bacterial invasion into gut-associated lymphoid tissues. ACS Infect. Dis..

[55] Gal-Mor O. (2019). Persistent infection and long-term carriage of typhoidal and nontyphoidal salmonellae. Clin. Microbiol. Rev..

[56] Kariuki S. (2006). Invasive multidrug-resistant non-typhoidal Salmonella infections in Africa: zoonotic or anthroponotic transmission?. J. Med. Microbiol..

[57] Demers-Mathieu V. (2018). Survival of immunoglobulins from human milk to preterm infant gastric samples at 1, 2, and 3 h postprandial. Neonatology.

[58] Lueangsakulthai J. (2020). Survival of recombinant monoclonal and naturally-occurring human milk immunoglobulins A and G specific to respiratory syncytial virus F protein across simulated human infant gastrointestinal digestion. J. Funct.Foods.

[59] Munoz-Quezada S. (2013). Competitive inhibition of three novel bacteria isolated from faeces of breast milk-fed infants against selected enteropathogens. Br. J. Nutr..

[60] Kantele A. (2012). Live oral typhoid vaccine Salmonella Typhi Ty21a - a surrogate vaccine against non-typhoid salmonella?. Vaccine.

[61] Maertens K. (2014). Breastfeeding after maternal immunisation during pregnancy: providing immunological protection to the newborn: a review. Vaccine.

[62] Rigsby P. (2020). Evaluation of a standardised Vi poly-l-lysine ELISA for serology of Vi capsular polysaccharide antibodies. Biologicals.

[63] Cerutti A. (2011). Regulation of mucosal IgA responses: lessons from primary immunodeficiencies. Ann. N. Y. Acad. Sci..

[64] Passali D., Bellussi L. (1988). Circadian changes in the secretory activity of nasal mucosa. Acta Otolaryngol..

[65] Otsuki T. (2004). Secretory IgA in saliva and academic stress. Int. J. Immunopathol. Pharmacol..

[66] Sojka M., Sayers A.R., Woodward M.J. (2001). Analysis of expression of flagella by Salmonella enterica serotype typhimurium by monoclonal antibodies recognising both phase specific and common epitopes. Vet. Microbiol..

[67] Ramachandran G. (2016). Functional activity of antibodies directed towards flagellin proteins of non-typhoidal Salmonella. PLoS One.

[68] Hiriart Y. (2013). Generation and selection of anti-flagellin monoclonal antibodies useful for serotyping Salmonella enterica. SpringerPlus.

[69] Li P. (2017). O-serotype conversion in Salmonella typhimurium induces protective immune responses against invasive non-typhoidal Salmonella infections. Front. Immunol..

[70] Winikoff B., Laukaran V.H. (1989). Breast feeding and bottle feeding controversies in the developing world: evidence from a study in four countries. Soc. Sci. Med..

[71] Turin C.G., Ochoa T.J. (2014). The role of maternal breast milk in preventing infantile diarrhea in the developing world. Curr. Trop. Med. Rep..

[72] Nachamkin I. (1994). Immunoglobulin A antibodies directed against Campylobacter jejuni flagellin present in breast-milk. Epidemiol. Infect..

[73] Shen J. (2018). No direct correlation between rotavirus diarrhea and breast feeding: a meta-analysis. Pediatr. Neonatol..

