# Supplementary material for: Complementary measurement of nontyphoidal Salmonella-specific IgG and IgA antibodies in oral fluid and serum
Source: Heliyon. 2022 Dec 15;9(1):e12071. doi: 10.1016/j.heliyon.2022.e12071 (PMC9871079; doi:10.1016/j.heliyon.2022.e12071)
Supplement: HLY_12071 - Supplementary file.docx [file mmc1.docx]

| Figure | Antigen | Ig | Origin | % Detectable Antibody | | | GMT | | | 95%CI (L/U) | | |
| --- | --- | --- | --- | --- | --- | --- | --- | --- | --- | --- | --- | --- |
|  |  |  |  | **UK Adults** | **Kenya Adults** | **Kenya Infants** | **UK Adults** | **Kenya Adults** | **Kenya Infants** | **UK Adults** | **Kenya Adults** | **Kenya Infants** |
| A | O:4,5 | IgG | Oral Fluid | 56%, 28/50 | 91.3%, 42/46 | 31.3%, 15/48 | 0.18 | 0.47 | 0.139 | 0.14/0.24 | 0.33/0.66 | 0.11/0.17 |
| B | O:4,5 | IgA | Oral Fluid | 98%, 49/50 | 100%, 45/45 | 79.2%, 38/48 | 4.99 | 10.57 | 6.27 | 3.89/6.4 | 8.29/13.49 | 4.21/9.34 |
| C | O:4,5 | IgG | Serum | 100%, 50/50 | 98%, 48/49 | 96%, 48/50 | 13.71 | 105.5 | 7.03 | 9.83/19.11 | 76.87/144.8 | 4.38/11.31 |
| D | O:4,5 | IgA | Serum | 100%, 50/50 | 100%, 49/49 | 74%, 37/50 | 15.37 | 54.45 | 3.35 | 11.43/20.68 | 41.47/71.49 | 2.04/5.51 |
| E | O:9 | IgG | Oral Fluid | 90%, 45/50 | 73.3%, 33/45 | 22.4%, 11/48 | 0.34 | 0.31 | 0.12 | 0.26/0.45 | 0.22/0.43 | 0.10/0.14 |
| F | O:9 | IgA | Oral Fluid | 100%, 50/50 | 91.1%, 41/45 | 79.2%, 38/48 | 12.3 | 14.29 | 10.06 | 9.76/15.52 | 9.59/21.3 | 6.4/15.82 |
| G | O:9 | IgG | Serum | 100%, 50/50 | 98%, 48/49 | 96%, 48/50 | 35.19 | 97.76 | 4.9 | 25.16/49.22 | 63.89/149.6 | 3.37/7.13 |
| H | O:9 | IgA | Serum | 100%, 50/50 | 98%, 49/50 | 70%, 35/50 | 23.13 | 63.13 | 4.7 | 18.51/28.9 | 43.58/91.45 | 3.25/6.78 |
| I | Flagellin STM | IgG | Oral Fluid | 92%, 46/50 | 100%, 46/46 | 81.3%, 39/48 | 0.28 | 0.21 | 0.17 | 0.22/0.39 | 0.17/0.27 | 0.11/0.25 |
| J | Flagellin STM | IgA | Oral Fluid | 100%, 50/50 | 88.9%, 40/45 | 93.8%, 45/48 | 19.73 | 7.18 | 8.42 | 15.98/24.37 | 5.39/12.45 | 5.7/12.45 |
| K | Flagellin STM | IgG | Serum | 100%, 50/50 | 100%, 50/50 | 95.6%, 44/46 | 38.94 | 38.46 | 12.42 | 31.80/47.7 | 30.70/48.17 | 7.92/19.46 |
| L | Flagellin STM | IgA | Serum | 98%, 49/50 | 98%, 49/50 | 54%, 27/50 | 20.62 | 29.51 | 3.66 | 15.8/26.91 | 22.94/37.98 | 2.29/5.85 |
| M | Flagellin SEN | IgG | Oral Fluid | 88%, 44/50 | 89.1%, 41/46 | 68.8%, 33/48 | 0.23 | 0.16 | 0.12 | 0.17/0.3 | 0.12/0.20 | 0.095/0.16 |
| N | Flagellin SEN | IgA | Oral Fluid | 100%, 50/50 | 97.8%, 44/45 | 89.6%, 43/48 | 15.07 | 12.23 | 8.7 | 12.08/18.8 | 9.2/16.26 | 5.74/13.19 |
| O | Flagellin SEN | IgG | Serum | 100%, 50/50 | 100%, 50/50 | 100%, 49/49 | 44.46 | 51.85 | 17.09 | 35.03/56.43 | 42.6/63.11 | 11.53/25.34 |

**Table S1**

*Detectability of NTS specific oral fluid and serum IgG and IgA, geometric mean titres and 95% CI in phase 1 UK adults, Kenyan adults and Kenyan infants.*

| Figure | Antigen | Antibody | % Detectable Antibody | | | | | GMT | | | | | 95%CI (L/U) | | | | |
| --- | --- | --- | --- | --- | --- | --- | --- | --- | --- | --- | --- | --- | --- | --- | --- | --- | --- |
|  |  |  | **Infants**  **1-12m** | **Y. Children**  **13m-4y 11m** | **O. Children**  **5-14y 11m** | **Adults**  **15-54y 11m** | **Elderly**  **55y+** | **Infants**  **1-12m** | **Y. Children**  **13m-4y 11m** | **O. Children**  **5-14y 11m** | **Adults**  **15-54y 11m** | **Elderly**  **55y+** | **Infants**  **1-12m** | **Y. Children**  **13m-4y 11m** | **O. Children**  **5-14y 11m** | **Adults**  **15-54y 11m** | **Elderly**  **55y+** |
| A | O:4,5 | IgG | 20.3%, 12/59 | 45.8%, 33/72 | 57.4%, 31/54 | 74.2%, 76/62 | 76.4%, 42/55 | 0.114 | 0.156 | 0.204 | 0.226 | 0.236 | 0.123/0.127 | 0.131/  0.185 | 0.158/  0.263 | 0.183/  0.278 | 0.19/  0.294 |
| B | O:9 | IgG | 37.3%, 22/59 | 38.9%, 28/72 | 55.6%, 30/54 | 72.6%, 45/62 | 72.7%, 40/55 | 0.122 | 0.144 | 0.175 | 0.238 | 0.236 | 0.109/0.136 | 0.123/  0.168 | 0.142/  0.22 | 0.186/  0.305 | 0.182/  0.306 |
| C | Flagellin STM | IgG | 61%, 36/59 | 76.3%, 55/72 | 75.9%, 41/54 | 85.5%, 53/62 | 85.5%, 47/55 | 0.071 | 0.094 | 0.103 | 0.132 | 0.147 | 0.062/0.082 | 0.078/  0.113 | 0.085/  0.124 | 0.104/  0.168 | 0.117/  0.185 |
| D | Flagellin SEN | IgG | 59.3%, 35/59 | 76.1%, 54/71 | 80.4%, 45/56 | 87.1%, 54/62 | 81.8%, 45/55 | 0.073 | 0.096 | 0.183 | 0.179 | 0.164 | 0.065/0.081 | 0.082/  0.113 | 0.137/  0.243 | 0.14/  0.229 | 0.125/  0.215 |
| E | O:4,5 | IgA | 76.3%, 45/59 | 56.9%, 41/72 | 63%, 34/54 | 90.3%, 56/62 | 90.9%, 50/55 | 5.595 | 1.889 | 2.337 | 6.319 | 3.762 | 3.74/  8.36 | 1.53/  2.34 | 1.82/  3.0 | 4.7/  8.49 | 2.9/  4.88 |
| F | O:9 | IgA | 84.7%, 50/59 | 80.5%, 58/72 | 81.5%, 44/54 | 93.5%, 58/62 | 94.5%, 52/55 | 11.430 | 3.246 | 3.680 | 11.570 | 6.463 | 7.4/  17.66 | 2.49/  4.23 | 2.77/  4.9 | 8.11/  16.51 | 4.96/  8.43 |
| G | Flagellin STM | IgA | 88.1%, 52/59 | 63.3%, 45/71 | 76.8%, 43/56 | 91.9%, 57/62 | 89.1%, 49/55 | 5.018 | 2.333 | 2.957 | 5.538 | 4.375 | 3.47/  7.26 | 1.85/  2.94 | 2.31/  3.78 | 4.02/  7.63 | 3.42/  5.6 |
| H | Flagellin SEN | IgA | 88.1%, 52/59 | 70.4%, 50/71 | 89.3%, 50/56 | 95.2%, 59/62 | 91%, 50/55 | 6.276 | 2.469 | 3.747 | 6.284 | 4.383 | 4.45/  8.86 | 1.96/  3.11 | 2.9/  4.84 | 4.69/  8.43 | 3.5/  5.49 |

**Table S2**

*Detectability of NTS specific oral fluid IgG and IgA, geometric mean titres and 95% CI across a cross section of age groups in the Kenyan phase 2 population.*

**Figure S1: A comparison of the impact of oral fluid IgG sample dilutions on the ability to detect a correlation with serum IgG.**

Antigens tested (**A**) O; 4,5, (**B**) O:9, (**C**) flagellin STM, (**D**) flagellin SEN. All figures shown represent the plots for 1:2 oral fluid dilution with Spearman’s Rank r and P values shown in blue. All samples below the 1:10 limit of detection were assigned a value at the limit of detection. The Spearman’s Rank r and P values were analysed separately for the 1:10 limit of detection and are shown above in red.

**
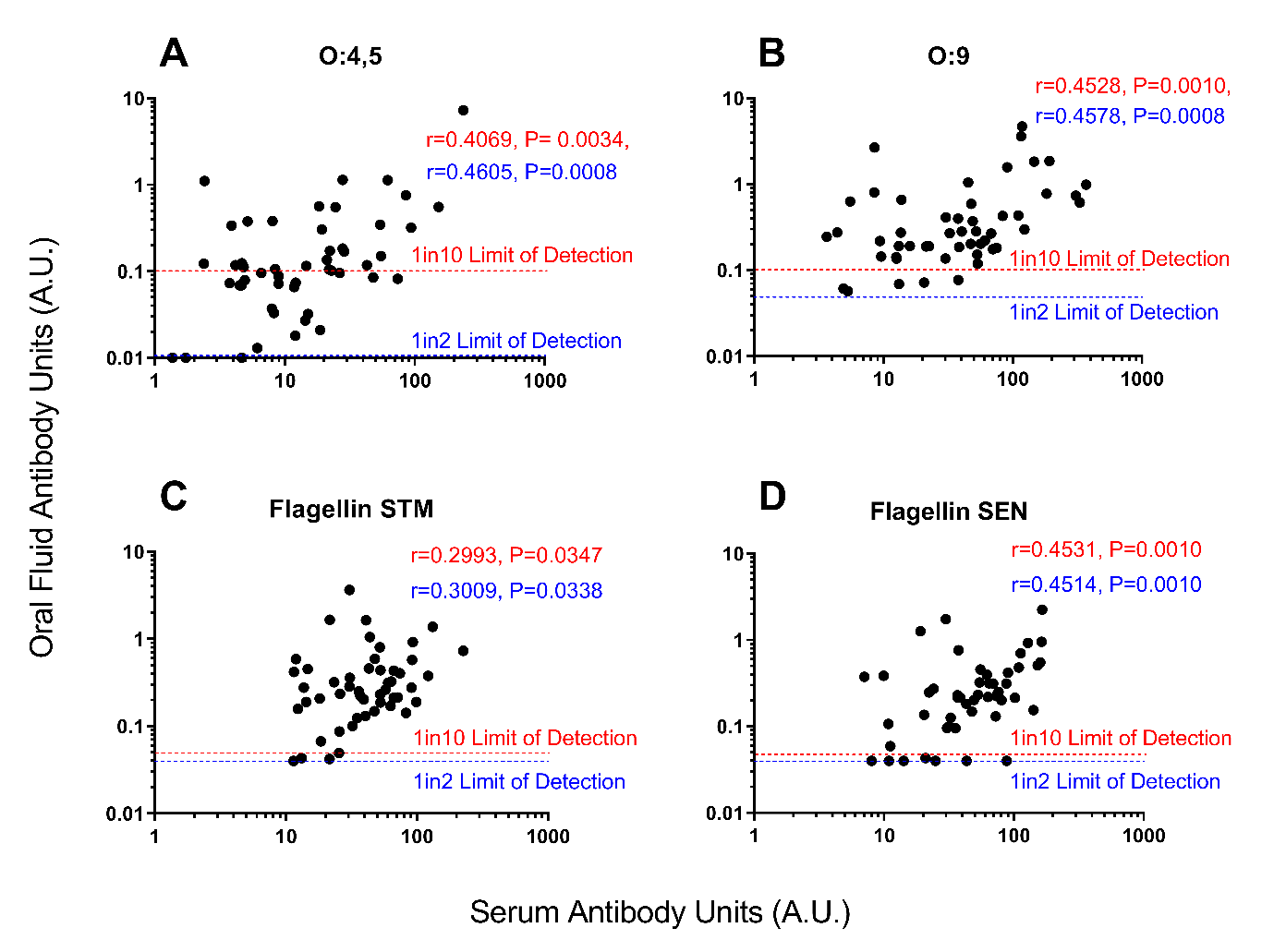
**
