# Supplementary figures and images for: Complementary measurement of nontyphoidal Salmonella-specific IgG and IgA antibodies in oral fluid and serum
Source: Heliyon. 2022 Dec 15;9(1):e12071. doi: 10.1016/j.heliyon.2022.e12071 (PMC9871079; doi:10.1016/j.heliyon.2022.e12071)

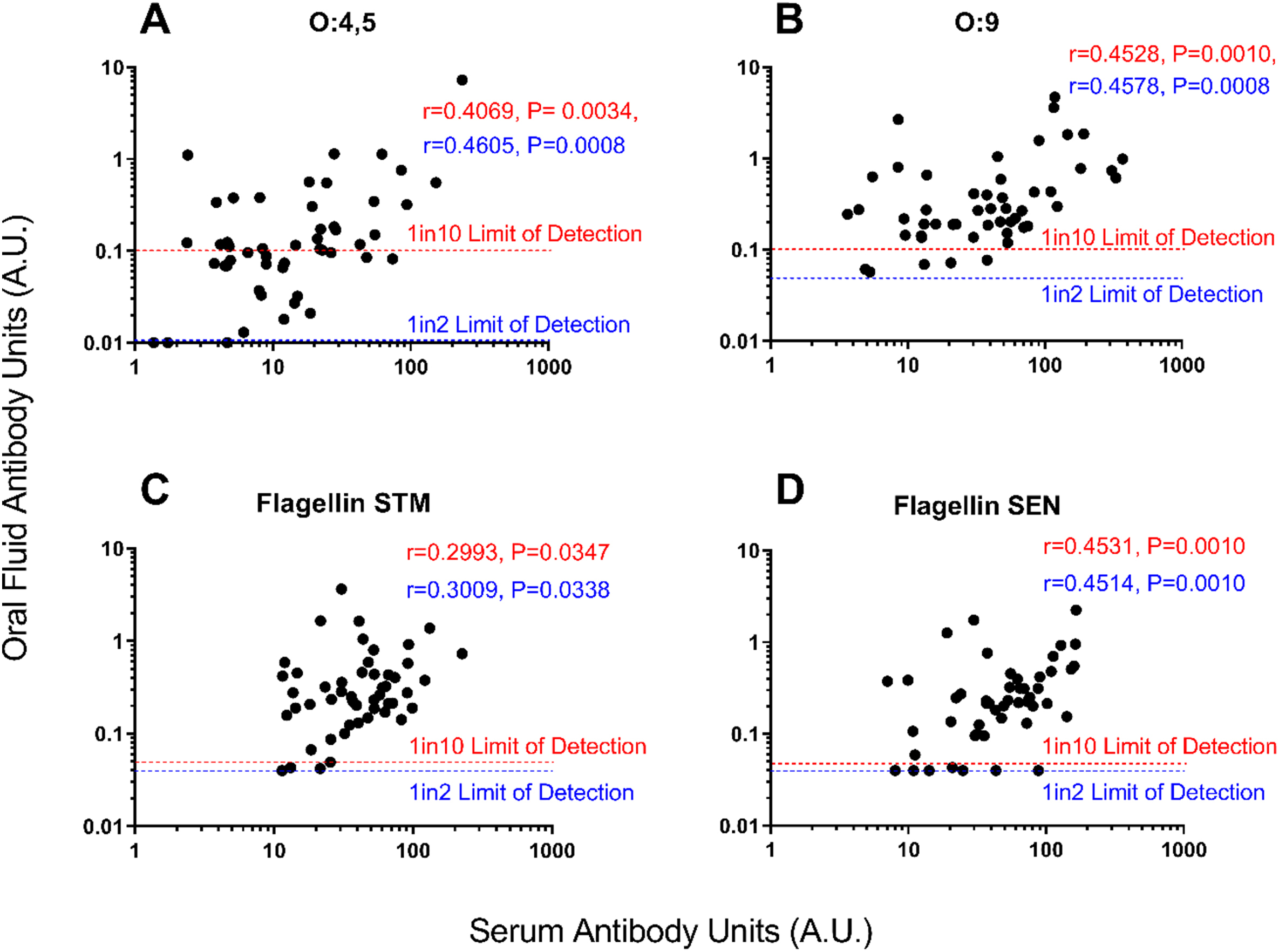

Supplement: Figure S1_V2.tif [file mmcfigs1.jpg]
